# Impact of psychoeducation on oncology-related knowledge and attitudes toward truth-telling, fairness, and beneficence in oncology care among medical residents from diverse specialties

**DOI:** 10.3389/fpsyg.2026.1850827

**Published:** 2026-08-10

**Authors:** Adelina Alcorta Garza, Oscar Vidal Gutiérrez, Javier Alejandro Martínez Moyano, Celia Beatriz González Alcorta, Fernando Alcorta Núnez, Mónica Lizeth Garza García, Paola Azucena López Sierra, Itzel Lidey Galaviz Reynoso, Aminta Mariel Cortés Almazán, Camila Alejandra Martínez Roque, Juan Francisco González Guerrero

**Affiliations:** 1 University Center Against Cancer, José E. González University Hospital, Universidad Autónoma de Nuevo León, Monterrey, Mexico; 2 Oncology Service, José E. González University Hospital, Universidad Autónoma de Nuevo León, Monterrey, Mexico

**Keywords:** attitudes toward oncological care, attitudinal change, medical residents, oncology knowledge, psychoeducation

## Abstract

**Background:**

Little is known about the effects of psychoeducational interventions on attitudes toward honest communication, equitable care, and acting in the cancer patient's best interests. We assessed the impact of a psychoeducational intervention on oncology-related knowledge and attitudes toward truth-telling, fairness, and beneficence in oncology care among medical residents from diverse specialties. We also examined factors associated with successful knowledge acquisition and changes in attitude.

**Methods:**

This was a quasi-experimental study with pre- and post-measures conducted at a public tertiary-care and teaching hospital. All residents were invited to participate, except those in the oncology specialty. The first measurement included 369 residents and the second 278 (loss rate = 24.7%). The intervention integrated psychological principles with educational strategies. It comprised either two 90-min sessions or 3 60-min sessions, delivered weekly or biweekly, and employed a range of psychoeducational techniques. Participants self-assessed their basic oncology-related knowledge using 7 questions (scored 0–100) and attitudes toward truth-telling, fairness, and beneficence in oncology care using 17 questions (scored 0–17). The effect size (ES) for repeated-measures designs was estimated to assess the magnitude of the observed change. A multivariate ordinal logistic regression analysis examined factors associated with knowledge acquisition and attitude change, categorized as successful, partially successful, or unsuccessful.

**Results:**

Mean oncology knowledge significantly increased following the intervention (51.8 ± 17.6 vs. 58.4 ± 17.7, *p* < 0.001; ES = 0.38). Being male was associated with lower odds of success (OR 0.55, 95% CI 0.31, 0.98), whereas training in a non–primary care–oriented specialty was associated with higher odds of success (OR 1.97, 95% CI 1.01, 3.84). Mean attitude also significantly improved following the intervention (12.3 ± 2.1 vs. 13.6 ± 2.0, p < 0.0001; ES = 0.64), and self-reported anxiety was associated with lower odds of successful attitude change (OR 0.41, 95% CI 0.24–0.71).

**Conclusions:**

The psychoeducational intervention was associated with moderate improvements in attitudes and modest improvements in oncology knowledge. The majority of residents still did not reach the desired level of knowledge, indicating that important educational gaps remained. Differences by sex, specialty orientation, and anxiety highlight the influence of learner characteristics on intervention outcomes.

## 1 Introduction

Cancer is one of the most common and deadly non-communicable diseases globally. In 2022, there were nearly 20 million new cases of cancer, resulting in an estimated 9.7 million cancer-related deaths (Bray et al., 2024). Doctors frequently need to deliver heartbreaking news, but they may encounter difficulties communicating prognoses in oncological care while adhering to ethical and moral principles, such as beneficence, justice, and professionalism (Bousquet et al., 2015; Baliga et al., 2024). Some may feel a strong resistance against the disclosure of diagnosis and prognosis in terminally ill patients (Sarafis et al., 2013). Moreover, they should provide sufficient information and address the concerns of cancer patients and their families within a limited consultation time (Gilligan et al., 2017). Residency is critical for acquiring skills before habits form, and residents face communication barriers due to emotional challenges and the complexities of cancer care (Rivest et al., 2023). Psychoeducation is an intervention modality that combines psychotherapeutic and educational strategies, drawing on various theories—such as ecological systems theory, cognitive-behavioral theory, and learning theory—as well as several models of practice, including group practice, stress and coping, social support, and narrative frameworks (Lukens and McFarlane, 2004). It has been associated with several beneficial outcomes, including improved disease knowledge and attitudes, increased adherence to diagnostic procedures and treatment, and reduced psychological distress (Barlow and Ellard, 2004; Lukens and McFarlane, 2004; Setyowibowo et al., 2022; Burke et al., 2024; Marks et al., 2024).

Traditionally, psychoeducationalinterventions have been used in the management of mental disorders such as schizophrenia, bipolar disorder, major depression, obsessive-compulsive disorder, substance use disorder, and borderline personality disorder (Sarkhel et al., 2020). However, their application has expanded to assist children and their families living with chronic diseases (Barlow and Ellard, 2004) and individuals with chronic communicable diseases (Burke et al., 2024) or breast cancer (Setyowibowo et al., 2022). It has also been recommended that psychoeducation become a standard of care in pediatric oncology (Thompson and Young-Saleme, 2015). Psychoeducation is not diagnosis-bound. It is defined by its mechanism, not by its diagnosis. Moreover, the psychological principles underlying psychoeducation—such as reflection, discussion, and emotional engagement—can also be applied in educational settings to promote learning and facilitate changes in attitudes and behaviors. Psychoeducational approaches that incorporate structured education, skill development, and cognitive reframing may help remove psychological barriers to processing complex and emotionally charged information and support the development of strategies for proactively using that information (Lukens and McFarlane, 2004). This conceptualization supports the use of a psychoeducational approach in oncology residency training. However, medical residents are rarely provided with opportunities to participate in psychoeducational training (Windsor, 2014).

Clinicians in oncology care must integrate technical knowledge with self-awareness and ethical attitudes while dealing with emotional barriers, particularly in contexts such as end-of-life care. However, a gap remains in what is known about medical residents' knowledge and attitudes toward truth-telling, fairness, and beneficence in oncology care. Furthermore, although psychoeducational interventions have been associated with improvements in knowledge, attitudes, and psychological coping, their application in residency training remains limited. Unlike traditional educational approaches, psychoeducation combines structured education with psychotherapeutic strategies such as self-reflection, cognitive reframing, and emotional processing, which may help residents overcome psychological barriers to communication and ethical decision-making. Nevertheless, its effects on medical residents‘ attitudes toward honest communication, equitable care, and acting in the best interests of cancer patients have not been well established.

This study assessed the impact of a psychoeducational intervention on oncology-related knowledge and attitudes toward truth-telling, fairness, and beneficence in oncology care among medical residents across clinical, surgical, and diagnostic specialties. Training programs that bring together physicians from multiple specialties may offer a richer learning environment, fostering a more holistic, adaptable, and collaborative approach to professional development. We also examined factors associated with successful knowledge acquisition and changes in overall attitude.

## 2 Materials and methods

**Figure 1:**
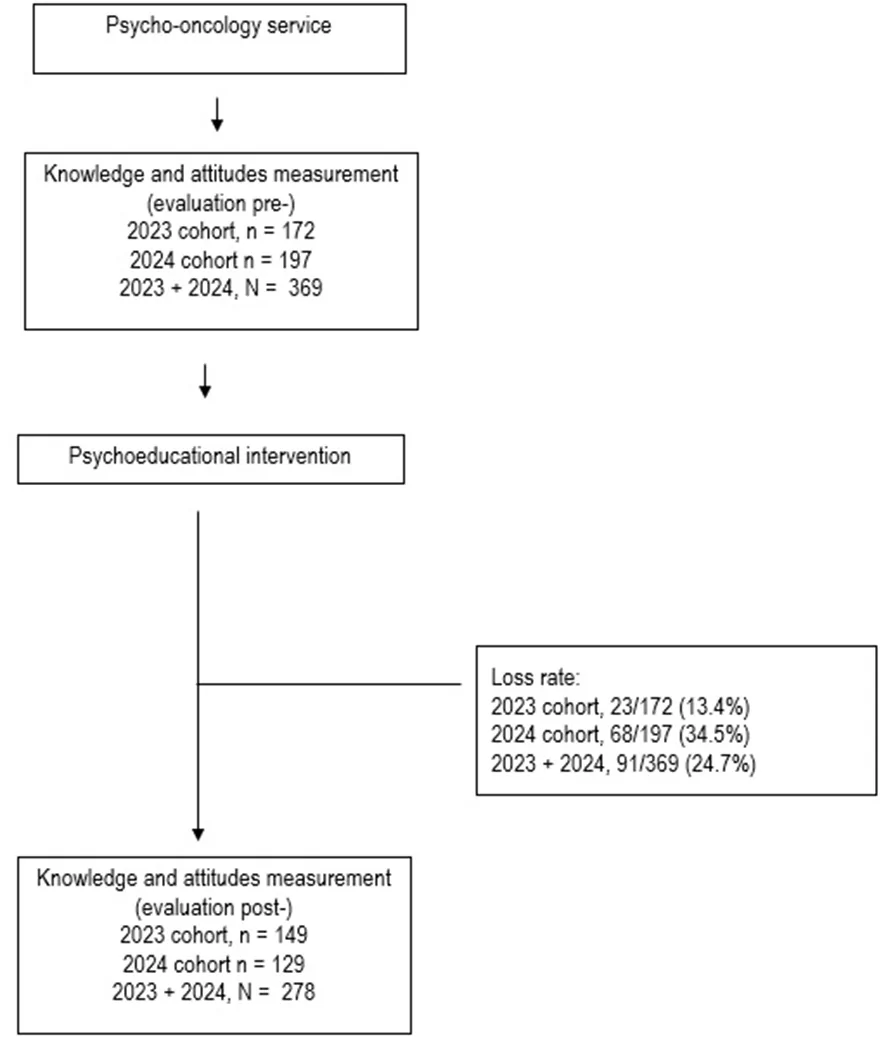
Study design.

This was a quasi-experimental study with pre- and post-measures conducted at a public tertiary-care and teaching hospital in Monterrey, Mexico, between 2023 and 2024 (Figure 1). The invitation was extended to all residents except those in the oncology specialty. No prerequisites were needed. The first measurement included 369 residents and the second 278, corresponding to a loss rate of 24.7%. The specialty frequency distribution remained similar before and after dropouts: clinical (69.6% vs. 69.7%), surgical (20.6% vs. 17.2%), and auxiliary-diagnostic (10.2% vs. 13.1%). Responders did not differ from non-responders in sex, marital status, family history of cancer, self-reported anxiety, or depression. However, responders were younger than non-responders (27.7 ± 2.7 vs. 28.4 ± 3.0, *p* < 0.03). A minimum sample size of 47 participants was estimated based on an effect size of 0.5 between two related populations, an alpha of 0.05, and a power of 0.95 (Faul et al., 2007). To account for a 30% loss rate, at least 61 participants were needed. All participants provided informed written consent.

### 2.1 Psychoeducational intervention

In the present study, psychoeducation was applied outside a psychopathological context. The intervention combined psychological principles with educational strategies to facilitate the acquisition of core oncology knowledge, promote emotional reflection, and change behavioral intentions. Several psychoeducational techniques were used to link intervention components to attitude change: (a) Focus groups incorporating projective techniques (e.g., hypothetical scenarios and role-based exercises) were utilized to reduce defensiveness and facilitate reflection. By externalizing attitudes toward truth-telling, fairness, and acting in the best interest of cancer patients, participants were encouraged to examine potential inconsistencies between professional ideals and clinical behaviors. Group discussion also allowed participants to observe and consider alternative perspectives, supporting reflective learning and potential cognitive reframing. (b) Art-based techniques were used to facilitate emotional engagement and reflective processing. Through symbolic and visual expression, participants were able to externalize experiences and values related to oncology practice, supporting deeper reflection and perspective-taking. (c) Expressive and reflective techniques were used to encourage self-expression and critical thinking on personal experiences and assumptions, promoting reflective practice and transformative learning regarding honest communication, fair, and beneficent oncology care. Learners were provided with core oncology concepts and essential information on the care of patients with cancer throughout the course of the illness. We also make participants aware of their current attitudes and how they affect their behavior and relationships with multidisciplinary team members involved in the treatment of cancer patients. The training consisted of 2 90-min sessions or 3 60-min sessions, delivered weekly or biweekly. The number of attendees per group ranged from 7 to 21. The sessions were led by a general psychiatrist with a master's degree in medical education, who practices psycho-oncology and has postgraduate training in bioethics. Medical oncologists reviewed the intervention's technical content. Participants answered a self-administered survey before joining the program (baseline measurement) and after the program's conclusion (7–14 days after the baseline measurement). Questionnaires were created using SurveyMonkey's electronic platform. A QR code was sent via WhatsApp for access.

### 2.2 Outcome measurements

#### 2.2.1 Oncology knowledge

Basic knowledge was assessed in Spanish using multiple-choice questions addressing the nature of cancer, multidisciplinary care, screening practices, and the roles of different specialties in cancer management (7 items). One item focused on the relatively recent development of medical oncology as a scientific discipline, an aspect considered part of disciplinary literacy. Correct responses were assigned a value of 1 and incorrect responses a value of 0 (see Supplementary Table 1), resulting in a raw score ranging from 0 to 7. Raw scores were subsequently transformed to a 0–100 scale using the following formula: (raw score/7) × 100. Higher scores indicate greater knowledge. Knowledge change was categorized as successful, partially successful, or unsuccessful (see Supplementary Table 1).

#### 2.2.2 Attitudes toward truth-telling, fairness, and beneficence in oncology care

Attitudes were assessed in Spanish using 17 items grouped into three domains. The items were developed and assigned *a priori* to the domains of truth-telling, fairness, and beneficence, based on expert consensus, to ensure broad coverage of ethically relevant principles in oncology care. Attitudes were conceptualized as evaluative dispositions toward honest communication and fair, beneficent oncology care. Reliability was evaluated using the Kuder–Richardson coefficient (KR-20), a measure of internal consistency for dichotomous items, which is considered equivalent to Cronbach's alpha for binary scales (Streiner et al., 2024). The domains included truth-telling (transparent communication with patients; 7 items, KR-20 pre- and post-intervention = 0.23 and 0.34, respectively), fairness (non-discriminatory clinical decision-making and care delivery; 5 items, KR-20 pre- and post-intervention = 0.38 and 0.40, respectively), and beneficence (acting in the patient's best interests and promoting supportive services throughout the cancer trajectory; 5 items, KR-20 pre- and post-intervention = 0.43 and 0.41, respectively). Participants were asked to select the statement that best reflected what they would do in the situation described. Responses indicating a favorable attitude were coded as 1 (see Supplementary Table 1). The total possible score ranged from 0 to 17; higher scores reflected a greater number of favorable attitudes. Attitude change was categorized as successful, partially successful, or unsuccessful (see Supplementary Table 2).

#### 2.2.3 Other variables

Sociodemographic data (sex, age, marital status, religion), family history of cancer (yes, no), self-reported anxiety or depression (yes, no), specialty type, and year of residence. Specialties were categorized as proposed by Hojat et al. (2005): (a) primary care–oriented, including disciplines focused on initial health and illness assessments, along with comprehensive episodic and longitudinal care for a broad range of medical conditions (e.g., family medicine). (b) non–primary care–oriented, including disciplines focused on providing episodic or longitudinal care for specific medical conditions (e.g., endocrinology). (c) technology-oriented, including disciplines focused on highly skilled and specialized therapeutic techniques or procedures (e.g., general surgery). (d) procedure-oriented, including disciplines primarily focused on specialized diagnostic procedures or applied laboratory work (e.g., nuclear medicine).

### 2.3 Statistical analysis

Descriptive statistics, means, and standard deviations for continuous variables and frequency distributions for categorical variables were used. The effect was analyzed using the *z*-test to compare two proportions (categorical variables) and the Wilcoxon test for related samples (continuous variables). The effect size (ES) for repeated-measures designs was estimated to assess the magnitude of the observed change (Hojat and Xu, 2004). A multivariate ordinal logistic regression analysis examined factors associated with knowledge acquisition and attitude change, categorized as successful, partially successful, or unsuccessful. Two models—one for knowledge and the other for attitude change—were independently executed using stepwise regression. Sociodemographic variables, specialty orientation, and self-reported anxiety and depression were included as independent variables, while family history of cancer was included as a control variable.

## 3 Results

The mean age was 27.9 ± 2.8. Most were female, had a partner, and identified as Catholic. Non-primary-care-oriented specialties were the most common, followed by primary-care-oriented, technology-oriented, and procedure-oriented. Most residents were in the first year of their degree (Table 1).

**Table 1:** Sociodemographic and degree-related information profile at baseline (*N* = 369).

Characteristic	*N*	%
Sex, female	192	52.2
Marital status, with a partner	335	91.0
Religion		
Agnostic/atheist	56	15.3
Catholic	280	76.5
Christian/Evangelist	30	8.2
Family history of cancer	107	29.1
Self-reported anxiety	138	37.5
Self-reported depression	75	20.4
Type of specialty		
Primary care oriented	102	27.6
Non-primary care	155	42.0
Technology-oriented	74	20.1
Procedure-oriented	38	10.3
Year of residency		
First	338	91.6
Second	31	8.4

**Figure 2:**
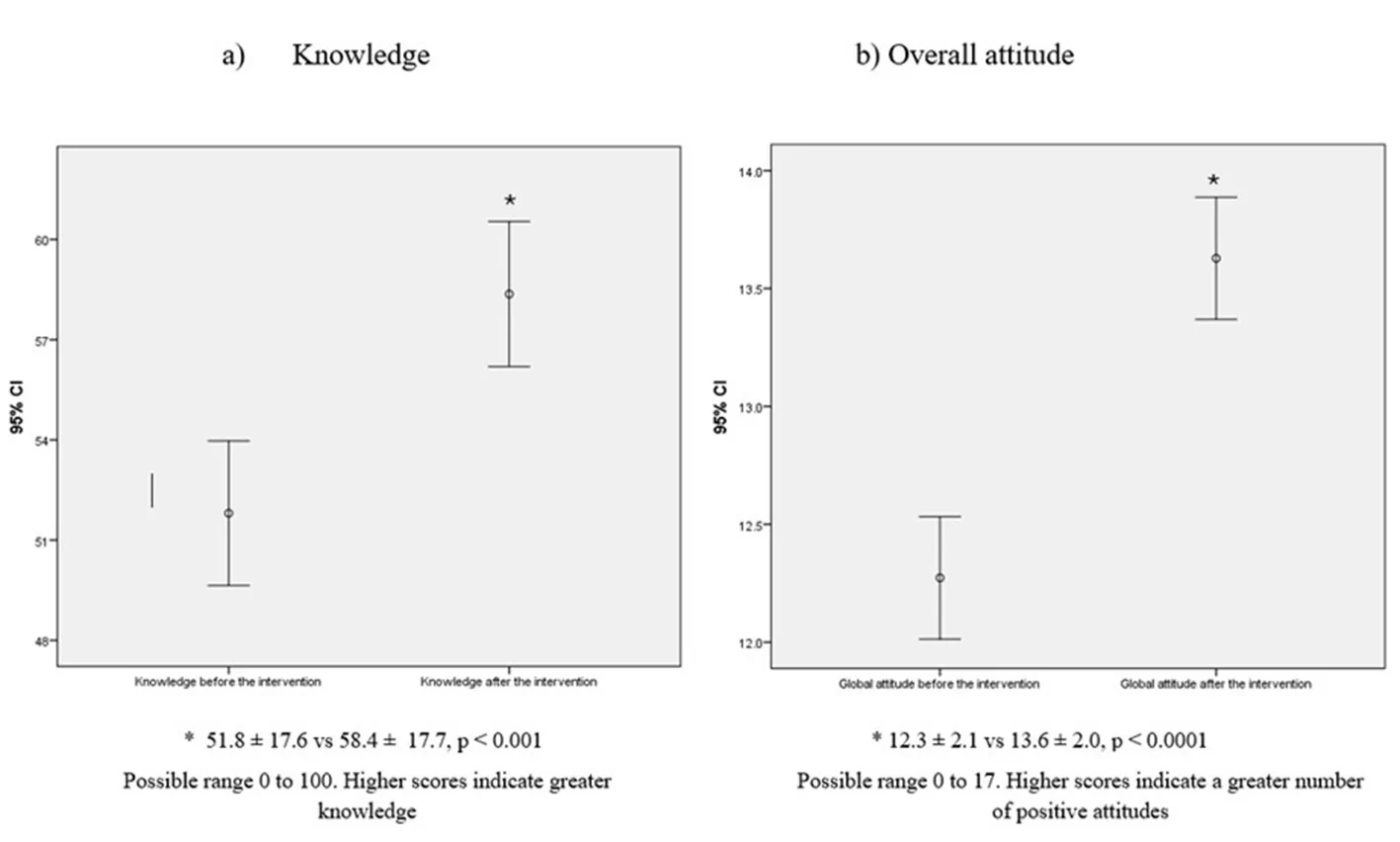
Knowledge and overall attitude before and after the psychoeducational intervention: **(a)** Knowledge; **(b)** Overall attitude.

**Figure 3:**
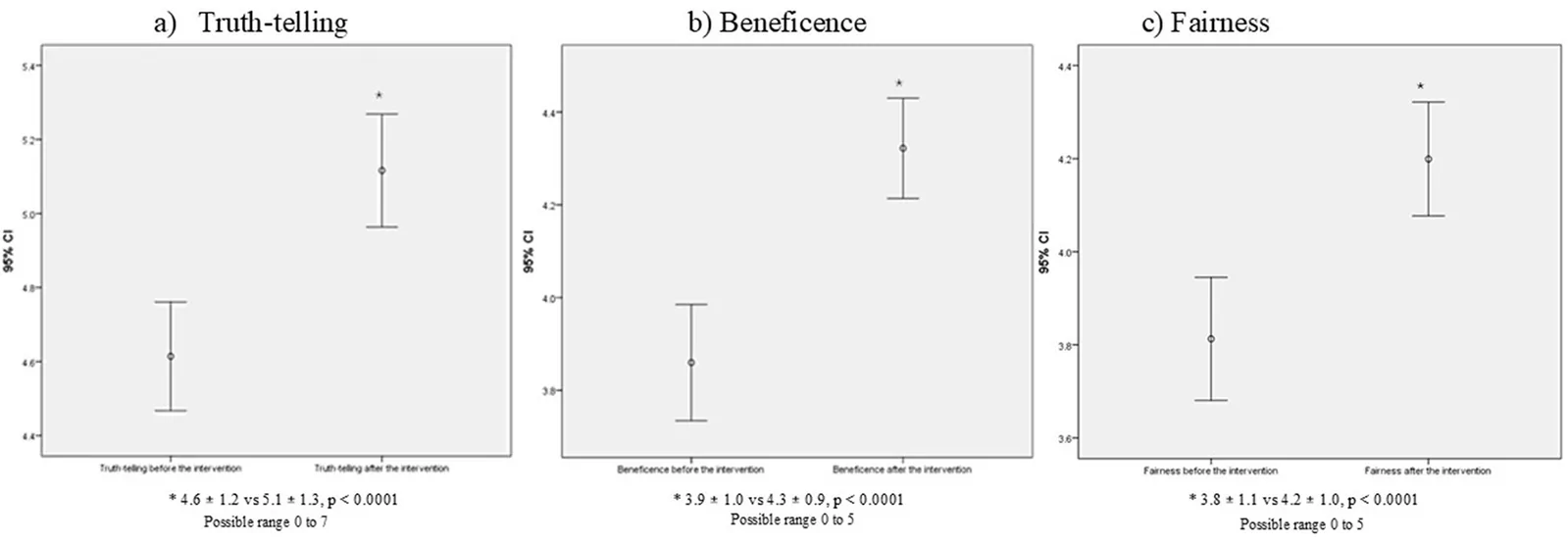
Attitude toward truth-telling, beneficence, and fairness before and after the psychoeducational intervention. **(a)** Truth-telling **(b)** Beneficence **(c)** Fairness.

The psychoeducational intervention significantly increased the mean knowledge and global attitude scores, with small and medium effect sizes of 0.38 and 0.64, respectively (*p* < 0.0001) (Figure 2). The improvement was significant across the attitude domains of truth-telling (ES = 0.42, *p* < 0.0001), beneficence (ES = 0.48, *p* < 0.0001), and fairness (ES = 0.35, *p* < 0.0001) (Figure 3). The frequency distributions for each item are shown in the supplementary files (See Supplementary Tables 3 and 4).

### 3.1 Factors associated with success

Knowledge acquisition outcomes were as follows: 16.0% achieved successful knowledge gain, 16.7% showed partial improvement, and 67.3% remained at low levels. Being male was associated with lower odds of success, whereas training in a non–primary care–oriented specialty was associated with higher odds of achieving or maintaining high scores. Overall attitude change outcomes were as follows: 58.7% achieved a favorable attitude, 17.4% showed partial improvement, and 24.0% remained at low levels. Self-reported anxiety was associated with lower odds of success (Table 2).

**Table 2:** Factors associated with success in knowledge acquisition and overall attitude change.

Characteristic	Knowledge outcome			OR	95% CI
	Unsuccessful	Partially successful	Successful		
Age 30+	24.9%	9.3%	26.8%	0.64	0.31, 1.32
Sex, male	49.4%	34.9%	36.6%	0.55	0.31, 0.98^*^
Marital status, without a partner	8.1%	11.6%	7.3%	1.19	0.45, 3.13
Religion, Christian/Evangelist	5.8%	7.0%	12.5%	0.99	0.56, 1.75
Type of specialty					
Primary care oriented	34.1%	25.6%	29.3%	Reference	
Non-primary care	28.9%	44.2%	43.9%	1.97	1.01, 3.84^*^
Technology-oriented	23.7%	14.0%	14.6%	0.73	0.32, 1.68
Procedure-oriented	13.3%	16.3%	12.2%	1.59	0.65, 3.89
Self-reported anxiety	38.2%	44.2%	39.0%	1.08	0.61, 1.92
Self-reported depression	20.2%	14.0%	22.0%	0.88	0.43, 1.81
Family history of cancer	33.5%	23.3%	24.4%	0.64	0.35, 1.18
Attitude outcome					
Age 30+	22.4%	23.8%	21.8%	0.89	0.46, 1.71
Sex, male	46.6%	52.4%	42.6%	0.72	0.42, 1.23
Marital status, without a partner	8.6%	7.1%	8.5%	0.66	0.24, 1.83
Religion, Christian/Evangelist	3.4%	2.4%	8.6%	0.91	0.51, 1.64
Type of specialty					
Primary care oriented	29.3%	33.3%	31.0%	Reference	
Non-primary care	27.6%	21.4%	42.3%	1.74	0.87, 3.48
Technology-oriented	31.0%	28.6%	13.4%	0.51	0.25, 1.04
Procedure-oriented	12.1%	16.7%	13.4%	1.03	0.43, 2.47
Self-reported anxiety	55.2%	45.2%	31.0%	0.41	0.24, 0.71^**^
Self-reported depression	24.1%	14.3%	20.4%	1.17	0.6, 2.31
Family history of cancer	34.5%	35.7%	28.2%	0.79	0.45, 1.38

* p < 0.05,** p < 0.001

## 4 Discussion

We assessed a psychoeducational intervention developed during medical residency to address the need for basic oncology knowledge and to foster favorable attitudes toward truthful communication, fairness, and beneficent care. One main distinction was the inclusion of medical residents from various clinical, surgical, and diagnostic specialties. The intervention increased basic oncology knowledge, although the estimated effect size was small. Moreover, the outcome categorization indicated that more than 6 out of 10 participants still needed further improvement. Inspection of specific topics revealed that the least favorable outcome was not due to clinical issues but to a historical one. The item concerning the length of time that medical oncology has been recognized as a scientific discipline yielded a relatively low proportion of correct responses. While medical oncology was formally recognized as a specialty fewer than 70 years ago, the scientific study of cancer has a much longer history. Consequently, respondents may have differed in their interpretation of the question, which may have influenced response patterns and should be considered when interpreting the results for this item. Besides, there is a need to strengthen general knowledge about cancer curability and cancer screening, especially breast cancer. Physicians must be agents of change and promoters of early cancer detection, regardless of their area of expertise. The success of knowledge acquisition is multifaceted and depends on internal (motivation, cognitive skills, sleep hours, and course attendance following a night shift) and external (learning strategies and techniques) factors (Vu et al., 2022; Dubinsky and Hamid, 2024). In this study, two factors were associated with successful knowledge outcomes. Male sex was associated with reduced odds of achieving or maintaining high knowledge scores. This finding may reflect differences in learning behaviors or engagement with the intervention and should be interpreted with caution; the underlying reasons warrant further investigation. In contrast, training in a non–primary care–oriented specialty was associated with higher odds of achieving or maintaining high knowledge scores. Although the underlying mechanisms were not directly assessed, this finding may reflect greater emphasis on disease-specific knowledge, technical training, and familiarity with oncology-related concepts in these specialties, compared with the broader, more generalist focus of primary care training.

Psychoeducation improved overall attitudes toward patient care, with a moderate effect size. The greatest change was observed in attitudes toward explicitly naming cancer, even when the patient exhibited anxiety. Nevertheless, nearly half of the participants continued to disagree with this statement following the intervention, suggesting that a single psychoeducational intervention may be insufficient to achieve substantial changes in this domain and highlighting the need for ongoing educational efforts. Previous research has similarly reported reluctance among healthcare professionals to disclose serious diagnoses and prognostic information. For example, (Awny et al. 2022) found that only 57.7% of physicians caring for or supervising terminally ill patients agreed that patients should be informed of their diagnosis, while 43.6% agreed that they should be informed of their prognosis. Communicating difficult information is a fundamental clinical skill; however, many practitioners report feeling inadequately prepared and express a need for additional training despite recognizing the importance of timely disclosure (Yousuf et al., 2024). Consistent with this need, educational interventions have been shown to improve both confidence and competence in breaking bad news among undergraduate and postgraduate physicians (Johnson and Panagioti, 2018). The GOCARE (Goals of Care Ambulatory Resident Education) course significantly increased internal medicine residents' self-rated confidence and willingness to engage in discussions about advanced care planning, including prognosis, bad news, treatment options, and hospice care (Berns et al., 2017). Also, a simulation followed by a briefing or a lecture-based intervention demonstrated significant improvement in self-perceived ability to deliver bad news and in empathy-building skills among gynecology and obstetrics residents (Karkowsky et al., 2016). Physicians differ significantly in their beliefs and practices regarding the disclosure of bad news about a patient's terminal condition, influenced by cultural values, ethical principles, and legal regulations. A Chinese study found that 66% of oncology clinicians would tell the truth if patients asked directly, while 10% would never disclose terminal conditions. Another 10% would usually do so even without a request (Wang et al., 2004). A study in Lebanon reported that only 19% of physicians routinely inform terminally ill patients about their diagnosis (Abu-Saad Huijer and Dimassi, 2007). This underscores the importance of researching attitudes across diverse populations.

Life expectancy is another sensitive topic for cancer patients. We found that almost 60% of participants had a positive attitude toward talking about their patients‘ remaining life after psychoeducation, compared to less than 45% before the intervention. The “Oncotalk” intervention reported that 81% of oncology fellows included an empathic statement or an exploratory question when the patient asked, “How much time do I have?,” compared to 52% before the intervention (Back, 2007). A review of studies on breaking bad news found that oncologists typically provide general time frames rather than precise timing because patients tend to misinterpret numbers. Providing patients with quantitative information helps clarify uncertainties, but it is important to acknowledge the limitations of applying statistics to individual cases (Bousquet et al., 2015). We identified that the proportion of participants with a positive attitude toward announcing the disease using the term “cancer,” rather than “mass” or “tumor,” remained stable at approximately 60% before and after the psychoeducational intervention. Back (2007) reported that oncology fellows' use of the word “cancer” when delivering bad news increased from 16% to 54%. Similarly, Williams et al. (2011) found that the use of the word “death” by first-year internal medicine residents during simulated end-of-life encounters rose from 17% to 83%. Oncologists often avoid terms like “death” and “cancer,” using more straightforward language and varying communication styles in directness, paternalism, and emotional emphasis (Bousquet et al., 2015).

### 4.1 Fairness

Fair care means impartiality, putting patients “needs first while maintaining high standards of competence and honesty (Baliga et al., 2024). In this study, the improvement in attitudes toward prioritizing patients' health needs and providing treatment regardless of cancer stage indicates that the intervention reinforced principles of equitable and patient-centered care. However, attitudes toward providing resuscitation without discrimination against cancer patients showed less improvement, suggesting that perceptions surrounding the appropriateness of aggressive or life-sustaining interventions in cancer patients may be more resistant to change. This may reflect the ethical complexity of end-of-life and critical care decisions, which could require more targeted educational approaches.

### 4.2 Beneficence

Palliative care is recognized as a fundamental component of the right to health and is most effective when integrated early in the disease trajectory. The observed improvements in attitudes related to cancer diagnosis disclosure and referral practices highlight the potential value of psychoeducational interventions in strengthening patient-centered communication. Participants agreed that the treating physician is often best positioned to communicate an initial cancer diagnosis because of their familiarity with the patient's clinical situation, emotional state, and personal circumstances. This role may facilitate a more supportive diagnostic experience and promote timely referral to oncology and psychosocial care services, both of which are essential components of comprehensive cancer care. The increased agreement regarding the need for palliative care evaluation suggests that psychoeducation may help reinforce the importance of integrating palliative care into routine cancer management. Early palliative care has been associated with reduced unnecessary hospital admissions and lower healthcare utilization while improving the quality of care. Despite these benefits, access to palliative care remains limited in many developing countries. Globally, an estimated 56.8 million people require palliative care each year, the majority of whom live in low- and middle-income countries. These findings underscore the importance of educational strategies that promote awareness of palliative care and its timely implementation. Only about 14% of people who need it currently receive it. Among the critical barriers to palliative care are limited awareness of healthcare professionals and misconceptions that it is only for those in their final weeks of life (World Health Organization, 2020). Therefore, medical practitioners' attitudes and practices are key in patient assessment and management.

Examination of associated factors with global attitude change showed that self-reported anxiety decreased the possibility of a successful outcome. Anxiety can disrupt cognitive functioning, emotional regulation, and social interactions. It often leads to avoidance behaviors and decreased self-confidence, making it difficult for individuals to maintain a positive attitude. Those experiencing anxiety may find it hard to concentrate, face challenges, or build healthy relationships. Anxiety must be addressed by utilizing coping strategies, seeking support, and pursuing treatment. A technology-oriented specialty was associated with a marginal reduction in the odds of the outcome. Surgical specialties are typically more focused on specific technical tasks rather than ongoing patient management. In contrast, clinically oriented specialties may place greater emphasis on communication and longitudinal care, fostering more positive, empathetic attitudes (Page, 2011; Rashid et al., 2021).

### 4.3 Study limitations

We have not yet examined the duration of the observed intervention effect. Future research should focus on evaluating the intervention's impact in the medium- and long-term. There was no control group. The research design was a one-group pretest-posttest study conducted among medical residents at a public tertiary-care teaching hospital. The findings might not be generalizable to residents receiving medical training in private and non-teaching hospitals. Because the sample consisted primarily of first-year residents (92%), caution is warranted when generalizing the findings to those at intermediate and advanced stages, whose attitudes may evolve with experience and training. Future studies should include a larger proportion of second- and third-year trainees to better examine developmental differences. We acknowledge that selection bias cannot be ruled out, as participation was voluntary. Individuals with more favorable attitudinal predispositions may have been more inclined to participate, potentially inflating the observed associations. Therefore, the findings should be interpreted with appropriate caution. We recognize that some items, such as “*There is no way to estimate how long a patient has left to live,”* may partially overlap with knowledge or beliefs in addition to attitudes. However, these items were classified by their primary intent to assess participants' value-based positions and clinical perspectives. The binary coding approach may not fully capture the complexity of clinical attitudes, which can be influenced by factors such as patient preferences, disease stage, and cultural context. However, because the participants were medical residents, the aim was to assess recognition of the most appropriate professional attitude in each scenario. Therefore, responses were coded dichotomously to facilitate the interpretation and comparison of pre- and post-intervention changes. Finally, we acknowledge the low internal consistency of the attitudinal measures, which fell below conventional acceptability thresholds. This finding may reflect heterogeneity in respondents” perspectives, as individuals may hold differing viewpoints or interpret item wording differently based on their experiences, backgrounds, and knowledge, resulting in varied response patterns. In the present study, the attitudinal domains were constructed to capture a broad range of expert-identified ethical principles rather than highly homogeneous latent constructs. Consequently, lower inter-item correlations may reflect the multifaceted and complex nature of the ethical concepts being assessed rather than measurement error alone. Nevertheless, the low internal consistency estimates warrant caution in interpreting the attitudinal findings and highlight the need for further research to evaluate and refine the instrument's measurement properties.

## 5 Conclusions

The psychoeducational intervention was associated with moderate improvements in attitudes and modest improvements in oncology knowledge across specialties. The majority of residents still did not reach the desired level of knowledge, indicating that important educational gaps remained. Differences by sex, specialty orientation, and anxiety highlight the influence of learner characteristics on intervention outcomes. The implementation of psychoeducational curricula in postgraduate medical residency programs should be prioritized to enhance cancer patient care.

## Data Availability

The raw data supporting the conclusions of this article will be made available by the authors, without undue reservation.
