# Supplementary material for: Impact of psychoeducation on oncology-related knowledge and attitudes toward truth-telling, fairness, and beneficence in oncology care among medical residents from diverse specialties
Source: Front Psychol. 2026 Aug 10;17:1850827. doi: 10.3389/fpsyg.2026.1850827 (PMC13500544; doi:10.3389/fpsyg.2026.1850827)
Supplement: Supplementary file 1 [file Supplementary_File_1.docx]

Additional File 1. Description of knowledge and attitudes items (question, answer

options, and rationale)(the assessments were administered in Spanish and

subsequently translated for the manuscript)

**KNOWLEDGE QUESTIONS** (participant should select the best and most complete answer)

| Question | Answer options | Rationale |
| --- | --- | --- |
| 1. What is Oncology? | Correct:  Oncology is the branch of medicine specializing in diagnosing and treating cancer. It includes medical oncology, radiation oncology, and surgical oncology.  Incorrect or incomplete options:  -A branch that focuses on the genetic understanding of cancer for the purpose of treatment; -A branch that studies cellular degeneration and cell death; -A branch that studies new drugs for cancer treatments. | Definition and scope of oncology |
| 2. What does medical oncology treatment include? | Correct:  It includes medical oncology, radiation oncology, surgery, psychiatry, nutrition, pain clinic, genetics, palliative care, and rehabilitation.  Incorrect or incomplete options:  -Medical Oncology, Radiation Oncology, and Surgery; -A medical oncologist and a surgeon are sufficient. | Awareness of the multidisciplinary nature of oncology care |
| 3. Which of the following diseases can be cured in some cases? | Correct: Lung cancer  Incorrect options:  -Diabetes mellitus; -Systemic hypertension; -Alzheimer's Disease; -Schizophrenia | Cancer prognosis: awareness that some cancers cases are potentially curable. |
| 4. In which of the following oncological diseases is screening performed? | Correct:  Breast, cervix, prostate, lung, colon, and esophagogastric junction.  Incorrect or incomplete options:  -Breast, cervical, prostate, lung, colon, and endometrial; -Breast, cervical, lung, and colon;  -Esophagogastric junction, colon, and lung. | Cancer screening: awareness of cancer screening programs |
| 7. What is the reason for requesting breast cancer self-examination? | Correct:  Help women become familiar with their bodies and be aware of breast cancer.  Incorrect options:  -Screening purposes; -To avoid seeing a doctor; -To promote firmness; -Self-stimulation | Awareness of the educational rather than screening role of breast self-examination. It is not recommended for screening purposes because by the time a tumor is large enough to be felt, it may already be too late for effective intervention |
| 5. What facts distinguish cancer from other diseases? | Correct:  Because it encompasses more than 200 diseases.  Incorrect options:  -It is a unique disease; -It is incurable, with a series of events that seem to respond to treatment but do not; -When it goes into remission, it means it wasn't cancer to begin with, or there was some kind of mistake. | Nature of cancer (awareness that cancer comprises a heterogeneous group of diseases) |
| 6. For how long has medical oncology been recognized as a scientific discipline? | Medical oncology is a discipline grounded in the scientific method, with fewer than 70 years of history  Incorrect option:  Just like all the other specialties | History of oncology (basic disciplinary knowledge) |

**ATTITUDE QUESTIONS** (participants were asked to select the statement that best reflected what they would do in the situation described)

| Question | Answer options | Rationale |
| --- | --- | --- |
| **Truth-telling (transparency when communicating with patients)** | | |
| 1. Should the doctor provide information about the progression to the metastatic stage, even if it causes sadness? | Favorable: It is better to provide information about the progression to the metastatic stage, even if it causes sadness  Unfavorable option: It is not better | It asks respondents to express a value-based position about truth-telling in emotionally difficult situations. Supports honest disclosure of disease progression despite potential emotional distress. |
| 2. Knowing that the patient's prognosis is poor… | Favorable: It is best to explain the prognosis to the patient, no matter how bad it is  Unfavorable option: It is not the best | It assesses respondents’ endorsement of honest communication and truth-telling in oncology care, particularly in difficult prognostic situations. Emphasizes transparency about prognosis. |
| 3. What word should be used when breaking the news about the disease? | Favorable: It is better to break the news of the disease by saying the word cancer, instead of “mass” or “tumor.”  Unfavorable option: It is not better | It evaluates respondents’ attitudes toward honest, direct communication with patients, specifically whether they endorse transparent disclosure rather than euphemisms. |
| 4. Should the doctor inform the patient about his/her cancer diagnosis, even if family members think otherwise? | Favorable: It is better to tell the patient the information about the cancer diagnosis, even if family members think otherwise  Unfavorable option: It is not better | It assesses respondents’ attitudes toward truth-telling, patients’ autonomy, and the patients’ right to information, especially when family preferences conflict with disclosure. Prioritizes direct disclosure to the patient, even if family members disagree. |
| 5. Should the doctor estimate how long the patient has left to live? | Favorable: It is best not to give an estimate when the patient asks how long he or she has left to live.  Unfavorable option: It is best to give an estimate so he/she can prepare for his/her death | It assesses respondents’ positions on prognostic disclosure and communication about end-of-life issues, rather than their factual knowledge. Physicians may avoid giving a precise estimate when survival predictions are highly uncertain to prevent misunderstandings, false expectations, or unnecessary distress (communication caution) |
| 6. What can be used to calculate a patient's lifespan? | Favorable: There is no way to estimate how long a patient has left to live  Unfavorable options:  -From an imaging study showing metastases; -Assessing the extent of treatment the patient has received: -Using evidence from a high-impact study with a population similar to the patient’s | It assesses attitudes toward communicating prognostic uncertainty in oncology care, rather than factual knowledge alone. Specifically, it reflects respondents’ positions regarding the limitations of estimating survival time and the role of truthful communication about uncertainty. |
| 7. What is the best way to call cancer when the patient shows anxiety? | Favorable: When a patient shows anxiety, the best way to call cancer is the term the patient prefers, as long as she/he agrees to treatment.  Unfavorable options:  -Malignant tumor; -Malignant mass; -Tumor; -Cancer | It assesses respondents’ endorsement of a particular communication approach in oncology care. Specifically, it reflects attitudes toward balancing truthful disclosure, emotional sensitivity, and patient-centered communication. |
| **Fairness (non-discriminatory clinical decision-making and care delivery)** | | |
| 1. A couple was involved in a motor vehicle accident, and both needed resuscitation maneuvers in the emergency room. He has a skull injury and a history of myocardial infarction, uncontrolled hypertension, and diabetes. She has an exposed bone fracture and a history of metastatic breast cancer. She arrived screaming in pain and suddenly went into cardiac arrest. To whom should resuscitation maneuvers be performed first? | Favorable: Between a patient with and one without cancer in the emergency room, priority should be given to those who present a change in alertness without discriminating against the patient with cancer.  Unfavorable options:  -The male patient should be treated first, as his condition has a better prognosis than the female patient’s; -The female patient should be treated first, as she suddenly experienced a change in her level of consciousness; -Given the prognosis for both patients, it is best to stop performing life-saving maneuvers; -The patient without cancer should be treated first, as the patient with metastatic cancer will die sooner or later anyway. | Emphasizes fair triage without discrimination, prioritizing patients according to clinical severity rather than diagnosis.  It assesses an attitude toward equitable and non-discriminatory care. |
| 2. Who should be treated first: a non-cancer patient in a hypertensive crisis in need of resuscitation or a cancer patient in need of resuscitation? | Favorable: Priority should be given to the one with the greatest need, without discriminating against the patient with cancer.  Unfavorable option: Priority should be given to the one without cancer | It reflects resuscitation priority based on clinical need rather than cancer status. It assesses an attitude toward equitable and non-discriminatory care. |
| 3. In cancer… | Favorable: All patients deserve ongoing care regardless of prognosis.  Unfavorable option: Not every patient should receive ongoing care | This item reflects attitudes toward equitable and continuous care by assessing respondents’ endorsement of the ethical principle that all patients with cancer deserve ongoing and appropriate care regardless of diagnosis, disease stage, or prognosis. |
| 4. Patients with metastatic cancer experiencing treatment-related toxicity… | Favorable: It is better for patients with metastatic cancer experiencing treatment-related toxicity to receive treatment in a hospital.  Unfavorable option: It is not better | It asks respondents to express a value-based position about  whether patients with metastatic cancer experiencing treatment-related toxicity deserve the same level of medical attention as patients with earlier-stage disease, regardless of disease stage. |
| 5. In a cancer patient… | Favorable: There will always be some kind of treatment; patients should continue to receive appropriate care regardless of disease stage.  Unfavorable option: There will not always be some kind of treatment | It reflects respondents’ endorsement of the ethical principle that patients deserve ongoing care regardless of prognosis. It assesses an attitude toward non-discriminatory care. |
| **Beneficence in oncology care (acting in the patient’s best interest and promoting supportive services throughout the cancer trajectory)** | | |
| 1. Oncology treatment… | Favorable: Oncology treatment is provided curatively in some cases and palliatively in others. Unfavorable option:  Oncology treatment is provided only if there is a chance of recovery | It reflects that attitudes toward supportive and palliative care are important components of cancer care throughout the disease trajectory, rather than factual knowledge alone. |
| 2. When is the best time for a cancer patient to be evaluated by a psycho-oncologist? | Favorable: The patient must be evaluated by psycho-oncology from the time of cancer diagnosis, without waiting for mental health symptoms to appear.  Unfavorable option: When signs or symptoms of a mental health condition appear | It assesses respondents’ endorsement of proactive involvement in psycho-oncology and supportive care throughout the cancer trajectory, rather than factual knowledge alone. |
| 3. When is the best time to evaluate a cancer patient for palliative care? | Favorable: The patient must be evaluated for palliative care from the time of cancer diagnosis.  Unfavorable option: Once there is no longer any treatment available | It assesses respondents’ endorsement of early integration of palliative care and supportive services throughout the cancer trajectory, rather than factual knowledge alone. |
| 4. Who should initially deliver the news of a cancer diagnosis? | Favorable: It is preferable for the treating physician to communicate the cancer diagnosis because they are familiar with the patient and their clinical history, and then refer the patient to an oncologist.  Unfavorable options:  -The oncologist; -The psychiatrist, since the news can cause a great deal of anxiety; -A family member, because they know the patient well and may help determine whether to disclose the diagnosis. | It asks respondents to express a value-based position by indicating whether they endorse the involvement of the treating physician in delivering the news |
| 5. Who must give the diagnosis of cancer to a patient? | Favorable: It is better for the treating physician to provide the initial cancer diagnosis and then refer the patient to an oncologist.  Unfavorable option:  It is better for the oncologist to provide the initial cancer diagnosis. | It asks respondents to express a value-based position by indicating whether they endorse the involvement of the treating physician in providing the initial cancer diagnosis |
