# Supplementary material for: Impact of psychoeducation on oncology-related knowledge and attitudes toward truth-telling, fairness, and beneficence in oncology care among medical residents from diverse specialties
Source: Front Psychol. 2026 Aug 10;17:1850827. doi: 10.3389/fpsyg.2026.1850827 (PMC13500544; doi:10.3389/fpsyg.2026.1850827)
Supplement: Supplementary file 2 [file Supplementary_File_2.docx]

Additional File 2. Classification of success in knowledge acquisition and attitude change

| Knowledge or attitude category ^a^ | | Outcome | Success category |
| --- | --- | --- | --- |
| Pre-intervention | Post-intervention |  |  |
| High/Very high | High/Very high | Remained high or very high, or changed from high to very high | Successful |
| Regular | High or very high | Improved | Successful |
| Low or very low | High or very high | Improved | Successful |
| Low or very low | Regular | Improved | Partially successful |
| Regular | Regular | Remained regular | Partially successful |
| Very Low/Low | Very Low/Low | Remained low or very low, changed from very low to low or vice versa | Unsuccessful |
| Regular | Low or Very Low | Worsened | Unsuccessful |
| Very high/High | Regular, Low, or Very Low | Worsened | Unsuccessful |

**^a^** Knowledge scores were categorized as follows: very high = 7, high = 6, average = 5, low = 4, and very low < 4. Attitude categories were defined as follows: very high = scores of 16–17; high = scores of 14–15; average = scores of 12–13; low = a score of 11; and very low = scores <11.
