# Supplementary material for: Impact of psychoeducation on oncology-related knowledge and attitudes toward truth-telling, fairness, and beneficence in oncology care among medical residents from diverse specialties
Source: Front Psychol. 2026 Aug 10;17:1850827. doi: 10.3389/fpsyg.2026.1850827 (PMC13500544; doi:10.3389/fpsyg.2026.1850827)
Supplement: Supplementary file 3 [file Supplementary_File_3.docx]

Additional File 3. Frequency distributions of participants with correct knowledge regarding basic oncology before and after the psychoeducational intervention.

|  | Evaluation pre- | | | Evaluation post- | | | Change |
| --- | --- | --- | --- | --- | --- | --- | --- |
| Item | n | N | % | n | N | % |  |
| 1. Oncology is the branch of medicine specializing in diagnosing and treating cancer. It includes medical oncology, radiation oncology, and surgical oncology. | 330 | 366 | 90.2 | 264 | 278 | 95.0 | +4.8* |
| 2. Lung cancer can be cured in some cases | 86 | 353 | 24.4 | 82 | 267 | 30.7 | + 6.3 |
| 3. Cancer is distinguished from other pathologies because it includes more than 200 diseases. | 232 | 363 | 63.9 | 186 | 270 | 68.9 | + 5.0 |
| 4. Oncology treatment includes medical oncology, radiation oncology, surgery, psychiatry, nutrition, pain clinic, genetics, palliative care, and rehabilitation. | 285 | 366 | 77.9 | 239 | 277 | 86.3 | +8.4 |
| 5. There is screening for different types of cancer, such as breast, cervix, prostate, lung, colon, and esophagogastric junction. | 257 | 367 | 70.0 | 185 | 277 | 66.8 | -3.2 |
| 6. The purpose of requesting a breast self-examination is to help women become familiar with their bodies and be aware of breast cancer. It is not intended for screening purposes (because by the time a tumor is large enough to be felt, it may already be too late for effective intervention). | 89 | 367 | 24.3 | 104 | 277 | 37.5 | + 13.3*** |
| 7. Medical oncology is a discipline grounded in the scientific method with fewer than 70 years of history | 52 | 367 | 14.2 | 66 | 275 | 24.0 | + 9.8** |

*p < 0.05, ** p < 0.01, *** p < 0.001 (z-test to compare the differences between two proportions)
