# Supplementary material for: Impact of psychoeducation on oncology-related knowledge and attitudes toward truth-telling, fairness, and beneficence in oncology care among medical residents from diverse specialties
Source: Front Psychol. 2026 Aug 10;17:1850827. doi: 10.3389/fpsyg.2026.1850827 (PMC13500544; doi:10.3389/fpsyg.2026.1850827)
Supplement: Supplementary file 4 [file Supplementary_File_4.docx]

Additional File 4. Frequency distributions of participants with a favorable attitude before and after the psychoeducational intervention.

|  | Evaluation pre- | | | Evaluation post- | | |  |
| --- | --- | --- | --- | --- | --- | --- | --- |
|  | n | N | % | n | N | % | Change |
| Truth‑telling |  |  |  |  |  |  |  |
| 1. It is better to provide information about the progression to the metastatic stage, even if it causes sadness | 362 | 366 | 98.9 | 273 | 278 | 98.2 | -0.7 |
| 2. It is best to explain the prognosis to the patient, no matter how bad it is | 348 | 365 | 95.3 | 268 | 278 | 96.4 | +1.1 |
| 3. It is better to break the news of the disease by saying the word cancer, instead of “mass” or “tumor.” | 223 | 366 | 60.9 | 175 | 278 | 62.9 | +2.0 |
| 4. It is better to tell the patient the information about the cancer diagnosis, even if family members think otherwise | 294 | 365 | 80.5 | 243 | 278 | 87.4 | +6.9* |
| 5. It is best not to give an estimate when the patient asks how long he or she has left to live. | 159 | 366 | 43.4 | 160 | 277 | 57.8 | +14.3*** |
| 6. There is no way of estimating how long a patient has left to live. | 166 | 366 | 45.4 | 155 | 277 | 56.0 | +10.6** |
| 7. It is best to call cancer by its name, even if the patient shows anxiety. | 126 | 362 | 34.8 | 144 | 276 | 52.2 | +17.4*** |
| Fairness |  |  |  |  |  |  |  |
| 1. Between a patient with and one without cancer in the emergency room, priority should be given to those who present a change in alertness without discriminating against the patient with cancer | 315 | 361 | 87.3 | 252 | 275 | 91.6 | 4.4 |
| 2. Between a patient with and one without cancer with a hypertensive crisis in need of resuscitation, priority should be given to the one with the greatest need without discriminating against the patient with cancer | 242 | 364 | 66.5 | 196 | 269 | 72.9 | 6.4 |
| 3. All patients deserve ongoing care regardless of prognosis | 279 | 365 | 76.4 | 242 | 275 | 88.0 | 11.6*** |
| 4. It is better for patients with metastatic cancer experiencing treatment-related toxicity to receive treatment in a hospital | 336 | 365 | 92.1 | 246 | 271 | 90.8 | -1.3 |
| 5. There will always be some kind of treatment for a cancer patient (patients should continue to receive appropriate care), regardless of disease stage. | 185 | 365 | 50.7 | 206 | 274 | 75.2 | 24.5*** |
| Beneficence |  |  |  |  |  |  |  |
| 1. Oncology treatment is provided curatively in some cases, while in others, it is offered palliatively | 360 | 367 | 98.1 | 272 | 275 | 98.9 | 0.8 |
| 2. The patient must be evaluated by psycho-oncology from the time of cancer diagnosis, without waiting for mental health symptoms to appear | 356 | 367 | 97.0 | 264 | 271 | 97.4 | 0.4 |
| 3. The patient must be evaluated for palliative care from the time of cancer diagnosis, without waiting until there is no longer a cure | 258 | 365 | 70.7 | 229 | 273 | 83.9 | 13.2*** |
| 4. It is better for the treating physician to provide the initial cancer diagnosis. | 202 | 365 | 55.3 | 204 | 278 | 73.4 | 18.0*** |
| 5. The treating physician must diagnose cancer because he/she has been in contact with the patient, and then he/she will refer him to the oncologist. | 237 | 367 | 64.6 | 222 | 277 | 80.1 | 15.6*** |

*p < 0.05, ** p < 0.01, *** p < 0.001 (z-test to compare the differences between two proportions)
